# A Ten-Minute Synthesis of α-Ni(OH)_2_ Nanoflakes Assisted by Microwave on Flexible Stainless-Steel for Energy Storage Devices

**DOI:** 10.3390/nano12111911

**Published:** 2022-06-02

**Authors:** Sumaih F. Alshareef, Nuha A. Alhebshi, Karima Almashhori, Haneen S. Alshaikheid, Faten Al-hazmi

**Affiliations:** Physics Department, Faculty of Science, King Abdulaziz University, Jeddah 21589, Saudi Arabia; salshareef0067@stu.kau.edu.sa (S.F.A.); kalhawwiti@stu.kau.sa (K.A.); heid0009@stu.kau.edu.sa (H.S.A.); fialhazmi@kau.edu.sa (F.A.-h.)

**Keywords:** nickel hydroxide, nanoflakes, microwave-assisted, hydrothermal, alpha phase, beta phase, supercapacitors, batteries, areal capacity, stainless steel

## Abstract

Although numerous methods have been widely used to prepare nickel hydroxide materials, there is still a demand for lowering the required heating time, temperature, and cost with maintaining a high-quality nanomaterial for electrochemical energy storage. In this research, we study the relationship between microwave-assisted heating parameters and material properties of nickel hydroxide nanoflakes and evaluate their effect on electrochemical performance. X-ray diffraction spectra show that the samples prepared at the highest temperature of 220 °C have crystallized in the beta phase of nickel hydroxide crystal. While the sample synthesized at 150 °C in 30 min contains both beta and alpha phases. Interestingly, we obtained the pure alpha phase at 150 °C in just 10 min. A scanning electron microscope shows that increasing the temperature and heating time leads to enlarging the diameter of the macro-porous flower-like clusters of interconnected nanoflakes. Electrochemical measurements in potassium hydroxide electrolytes demonstrate that the alpha phase’s electrodes have much higher capacities than samples containing only the beta phase. The maximum areal capacity of 17.7 µAh/cm^2^ and gravimetric capacity of 35.4 mAh/g are achieved, respectively, at 0.2 mA/cm^2^ and 0.4 A/g, with a small equivalent series resistance value of 0.887 ohms on flexible stainless-steel mesh as a current collector. These improved nickel hydroxide electrodes can be ascribed to utilizing the diffusion-controlled redox reactions that are detected up to the high scan of 100 mV/s. Such fast charge-discharge processes expand the range of potential applications. Our nickel hydroxide electrode, with its rapid preparation at medium temperature, can be a cost-effective candidate for flexible supercapacitors and batteries.

## 1. Introduction

Recently, the demand for electrical energy storage devices has rapidly increased in several global industries, such as electric vehicles, portable electronics, and renewable energy systems. As a consequence, the development of energy storage devices has become crucial. Supercapacitors, which are also known as electrochemical capacitors, are considered to be one of the most promising energy storage devices due to their high power density, long cycle life (>10^6^ cycles), broad operating thermal range (from −40 to 70 °C), low cost, flexible packaging, and excellent reversibility [1,2]. Supercapacitors are currently used in different applications such as backup power, solar power, regenerative braking, flashlights, and burst-mode power delivery within vehicles such as trains, cars, and cranes.

According to their charge storage mechanisms, supercapacitors can be classified into electrical double-layer capacitors (EDLCs), pseudocapacitors (PSCs), and hybrid supercapacitors. A Faradaic mechanism in PSCs involves fast and reversible reduction–oxidation (redox) reactions to transfer charges. Such electrochemical reactions store more charges than the electrostatic process of EDLCs [3]. Conway was the first to define pseudocapacitance to characterize materials with electrochemical signatures similar to EDLCs, but with a distinct charge storing mechanism [4]. Additionally, the PSCs behave like batteries in that they store charges by chemical reactions on cathodes and anodes, and ions are transferred between the electrodes via an electrolyte. However, the primary distinction between batteries and pseudocapacitors is that pseudocapacitors’ charging/discharging behavior happens on the surface or near-surface of the electrode materials in seconds and minutes. In batteries, the energy is stored in the entire bulk of electrode materials in hours [5,6].

Transition metal oxides/hydroxides and conducting polymers are good examples for the electrodes of PSCs [7]. Among transition metal oxide/hydroxide materials, ruthenium dioxide (RuO_2_) is a well-studied capacitive material due to its high electrical conductivity, large potential window (greater than 1 V), and numerous oxidation states. The pseudocapacitance of RuO_2_ supercapacitor was first described by Trasatti and Buzzanca [8]. However, its scarcity and toxicity hinder its use on a large scale. Alternatively, other low-cost candidates for pseudocapacitors are attracting more interest, such as manganese oxides (MnO_2_) [9,10] and iron oxide/hydroxides [11]. Meanwhile, nickel oxide/hydroxides have been extensively researched and evaluated as potential battery-type materials for hybrid supercapacitors due to their high theoretical capacities, thermal and chemical stabilities in various electrolytes, and simple fabrication protocols [12]. The charge storage mechanism of nickel oxide (NiO) is mostly based on surface adsorption and redox reaction with hydroxide ions (OH¯). In contrast, the nickel hydroxide (Ni(OH)_2_) mechanism involves the intercalation and deintercalation of H^+^ between the layers. Because H^+^ has a smaller size than OH^−^, it is easier for H^+^ to diffuse into Ni(OH)_2_, giving Ni(OH)_2_ greater bulk characteristics than NiO [13]. Furthermore, Ni(OH)_2_ has produced considerably higher specific capacity values than NiO [14].

The history of nickel hydroxide as an electrode dates back to the nineteenth century when it was used as an electrode material for battery technologies [15]. However, nickel hydroxides are no longer restricted to the energy storage field. This material is now used in a wide range of practical applications, such as photocatalysis [16], electrocatalysis [17], electrochemical sensors [18], and many more. There are two basic nickel hydroxide structures, which are α-Ni(OH)_2_ and β-Ni(OH)_2_. Aqueous alkaline solutions, such as potassium hydroxide (KOH), are extensively used electrolytes for nickel hydroxide. The structure of nickel hydroxide materials significantly impacts electrochemical performance [19,20]. During the charging process in the KOH electrolyte, β-Ni(OH)_2_ with an oxidation state of +2 converts to β-NiOOH with an oxidation state of +3, while α-Ni(OH)_2_ is oxidized to a higher valance of +3.6 in γ-NiOOH [21,22]. Therefore, the α-Ni(OH)_2_ has a higher theoretical electrochemical capacity than that of β-Ni(OH)_2_ [22] which was confirmed experimentally as well as reported by Hu and Noréus, for example [23]. However, α-Ni(OH)_2_ is an unstable phase that tends to convert to β-Ni(OH)_2_ in alkaline electrolytes or when subjected to charge-discharge cycles [21,23,24]. Moreover, nickel cathode is converted to nickel oxide and nickel oxyhydroxide during the operation with zinc anode in KOH, as reported by Gerasopoulos et al. [25]. Their nickel–zinc microbatteries reach a maximum areal capacity of 4.5 µAh/cm^2^ at 0.05 mA/cm^2^ with a footprint area of 0.64 cm^2^. The fabrication protocols in that work involved photolithography and electroless plating on a silicon wafer, which is not convenient for the emerging flexible electronics. For hybrid supercapacitors, nickel aluminum layered double hydroxide (NiAl-LDH) on a copper foam was utilized as a battery-type electrode by Sekhar et al. [26]. The electrode was synthesized at 90 °C for 24 h and exhibited an areal capacity of 8.3 µAh/cm^2^ at 25 mA/cm^2^. When cobalt hydroxide was added to the NiAl-LDH electrode, the areal capacity promoted to 208.3 µAh/cm^2^ at the same current density, as the mass loading increased from 0.61 to 0.85 mg/cm^2^. Notably, the required co-precipitation time of the aforementioned NiAl-LDH is 24 h in an oven at 90 °C, which is considered a long time.

There are already hundreds of known methods for synthesizing nickel hydroxide materials, including chemical precipitation [27], solvothermal [28], electrodeposition [29], sol-gel [30], and newer methods such as microwave-assisted synthesis [31] and sonochemistry [32]. Microwave-assisted synthesis is an easy process for producing micro/nano-sized materials. Microwave heating has several advantages, including enhanced reaction rate, rapid volumetric heating, improved product quality, decreased reaction times, high yield, energy saving, morphology controllability, and so on [33]. Additionally, microwave-assisted methods create nanoparticles with relatively consistent morphologies and small size distributions with high purity [34]. Microwave heating differs fundamentally from traditional heating in that thermal energy is given to the material’s surface via radiant or convection heating and then transmitted to the bulk of the material via conduction. On the other hand, microwave energy is given directly to the material’s volume via molecular interaction with the electromagnetic field [35]. As a result, thick materials can be heated uniformly and quickly. Nickel salts and alkaline solutions have been commonly used in homogeneous precipitation methods for the nucleation and growth of Ni(OH)_2_ [36]. During the microwave-assisted synthesis, the dissolved urea in water undergoes thermal hydrolysis and reacts with hexaaquo Ni^2+^, condensing in Ni_4_(OH)_4_^4+^ then Ni(OH)_2_ [37]. Multiple publications have reported the microwave synthesis of nickel hydroxides with various nanostructured configurations and morphologies. For example, Zhang et al. [38] synthesized three-dimensional flower-like α-Ni(OH)_2_ nanostructures using microwave-assisted reflux in 30 min at 80 °C. In addition, Elshahawy et al. [39] reported the synthesis of β-Ni(OH)_2_ nanoparticles by microwave-assisted hydrothermal technique in 30 min at 150 °C, utilizing cetyltrimethylammonium (CTAB) as a surfactant. 

In the current work, a pure alpha phase of nickel hydroxide nanoflakes was optimized in only 10 min of microwave-assisted heating at 150 °C without surfactants or additives. Its structural and morphological properties are analyzed and compared with beta and mixed phases of nickel hydroxides prepared in different heating times and temperatures. We investigated the effects of these properties on the electrochemical performance of Ni(OH)_2_-based electrodes in KOH electrolytes. Additionally, flexible stainless-steel mesh was used as a current collector, minimizing the device’s resistance. Our flexible electrodes, with their fast preparation at medium temperature, can be a cost-effective candidate for hybrid supercapacitor and battery applications.

## 2. Materials and Methods

### 2.1. The Synthesis Method

The electrode materials in our work were synthesized by the microwave-assisted hydrothermal method. We experimentally set up the synthetic parameters of heating time and temperature to prepare a group of different samples, as abbreviated in Table 1.

Initially, a nickel salt of 7.47 g nickel acetate tetrahydrate (Ni(CH_3_CO_2_)_2_·4H_2_O, ACROS ORGANICS, Gee, Belgium) was dissolved in deionized water (25 mL) and 1.2 g urea (CO(NH_2_)_2_, Honeywell Riedel-de Haën AG, Seelze, Germany). All of them were stirred to obtain a clear green solution. This mixture was sealed into many Teflon-lined autoclaves for the microwave furnace with a power of 700 W (Ethos 1 Advanced Microwave Digestion System, Milestone Srl, Milan, Italy). For the first sample, the vessels were heated at 220 °C for 90 min. This experiment was repeated to prepare the other samples by changing the heating temperature and time as the following: 220 °C in 30 min, 150 °C in 30 min, and 150 °C in 10 min. After that, the solutions were transferred to centrifuge tubes with adding distilled water and cleaned in centrifugation for 5 min at 6000 rpm. The washing step was repeated using acetone and ethanol. Then, the solutions were filtered through a filter paper, followed by drying at 80 °C for 3 h. Finally, the precipitate was crushed to produce a fine green powder. The centrifugation cleaning steps are essential to remove the unreacted products.

### 2.2. Characterization Techniques

For the crystal structure characterization of our powder samples, an X-ray diffractometer (XRD) equipped with a Cu Kα X-ray source (λ = 1.5406 A°) was used (ULTIMA IV Advance XRD System, Rigaku, Tokyo, Japan). The crystallite size was calculated by using Sherrer’s formula (1).

(1) $$L=\frac{K\;\lambda }{B\;\cos\theta }$$

where (*L*) is a crystallite size (nm), (*K*) is a constant linked to crystallite shape (mostly assumed *K* ≈ 0.9), (*λ*) is the wavelength of the X-ray (nm), and (*B*) is the full width at half maximum (FWHM) of the peak (in radians). (*θ*) can be in radians or degrees because the (*cos θ*) corresponds to the same number. In addition, a field emission scanning electron microscope (FE-SEM) and energy dispersive X-ray spectroscopy (EDS) were used to obtain information about the elemental composition, surface morphology, and thickness. The SEM models used in this work were Teneo (ThermoFisher Scientific, Waltham, MA, USA) and JSM-7500F (JEOL, Tokyo, Japan). The microscopic images were generated by the secondary electrons from the specimen at small and large magnifications.

### 2.3. Electrochemical Measurements

We used flexible stainless steel (SS) mesh as a current collector (Type 316, Fuel Cell Store, College Station, TX, USA). It had a mesh size of 90 × 90, a wire diameter of 88.9 µm, an open area of 47%, and an open space between parallel wires of 193.04 µm. To attach the electrode material to the current collector substrate, the polyvinyl alcohol (PVA) binder was dissolved in DI water and stirred at under 70 °C. Then, the precipitated Ni(OH)_2_ powder was added to the mixture in Ni(OH)_2_:PVA weight ratio of 80:20, cast by drop onto a geometric area of 1 cm^2^ of the SS mesh at 60 °C. Finally, it was dried at room temperature overnight. The mass of the SS piece was weighted before and after the drop-casting process to obtain 0.5 mg of the deposited electrode materials.

A platinum (Pt) wire, saturated calomel electrode (SCE), and our Ni(OH)_2_-based electrode were used as a counter, reference, and working electrodes, respectively, in a three-electrode mode of electrochemical workstation (CHI 660D, CH Instruments, Austin, TX, USA). An aqueous solution of 3 M KOH was utilized as an electrolyte at room temperature. To evaluate the performance of our supercapacitor electrodes, electrochemical techniques including cyclic voltammetry (CV), chronopotentiometry (galvanostatic charge–discharge) (GCD), and electrochemical impedance spectroscopy (EIS) were conducted. The CV measurements were carried out at different scan rates ranging from 10 to 100 mV/s. The GCD techniques were used to calculate the capacity of a battery-type electrode by the following equation:

(2) $$Q=\frac{I\;\Delta t}{3600}$$

where (*Q*) is the capacity in ampere-hour (Ah), (*I*) is the set current in ampere (A), (Δt) is the discharging time in second (s), Areal and gravimetric capacities were calculated by dividing over the electrode area in squared centimeter or the mass loading in gram, respectively. The EIS measurements were conducted by applying an oscillation voltage of 5 mV with a frequency range of 0.1 Hz to 100 kHz, displayed in Nyquist plots.

## 3. Results and Discussion

### 3.1. Structural and Morphological Properties

**Figure 1:**
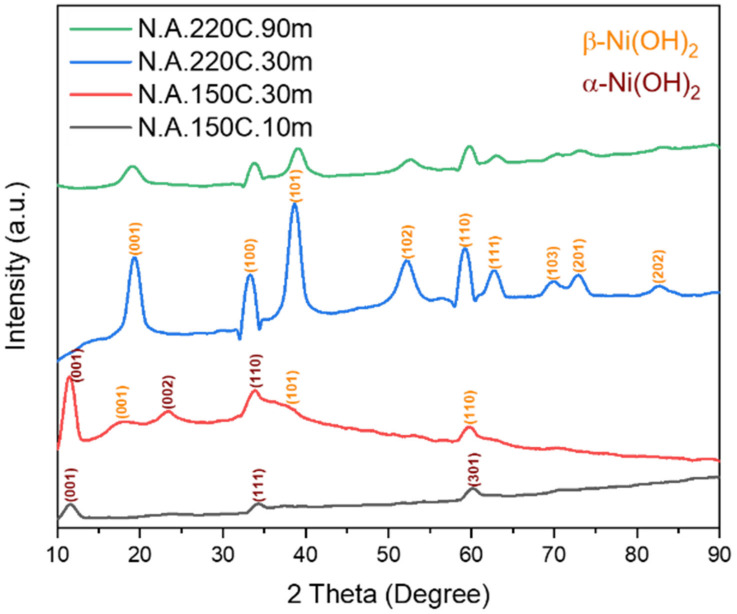
XRD patterns of Ni(OH)_2_ powder prepared at different heating times and temperatures.

The XRD patterns of the nickel acetate-derived samples are presented in Figure 1. For the sample prepared at 150 °C in 10 min (N.A.150C.10m), a pure α-phase of Ni(OH)_2_ is indicated by its characteristic atomic planes of (001), (111), and (301) at 11.6°, 34.3°, and 60.2°, respectively (JCPDS card no. 22-0444). As the heating time increases from 10 min to 30 min at the same temperature, new diffraction peaks are detected with a slight shift in the peak positions of α-(002), β-(001), and β-(101), representing that N.A.150C.30m consists of double-phase α/β nickel hydroxide. Such mixed-phase materials are recommended for electrodes due to the multiple redox reactions that store electrochemical energy [40]. When the heating temperature is raised from 150 °C to 220 °C, all the diffraction peaks are indexed as pure β-Ni(OH)_2_ (JCPDS card no. 14-0117), as reported in a previous article [39]. Finally, by increasing the heating time from 30 min to 90 min at 220 °C, the sample retains the β-phase with a slight shift in all peak positions except for (001) and (111). These shifts are attributable to bigger or smaller interlayer spacings occurring from differences in the quantities and coordination of water [41]. Additionally, the peaks become broader and lower, which indicates that the crystallinity is weaker than N.A.220C.30m. Although that β-Ni(OH)_2_ is thermodynamically more stable than the α-Ni(OH)_2_ [21,23,24], the latter has higher electrochemical capacities theoretically and experimentally [22,23], as explained in the introduction. Based on that, the N.A.150C.10m is expected to be a promising electrode candidate.

The crystallographic structure, including atomic planes (Miller indices), diffraction angles (2-theta), interlayer spacing (d-space), and average crystallite sizes of all nickel acetate-derived samples, are listed in Table 2. For the samples prepared in 30 min, there is a growth of the average crystallite size from 3.60 nm to 4.70 nm as the heating temperature increases from 150 °C to 220 °C. In contrast, it can be noticed that increasing the heating time at a fixed temperature leads to a decrease in the average crystalline sizes.

**Figure 2:**
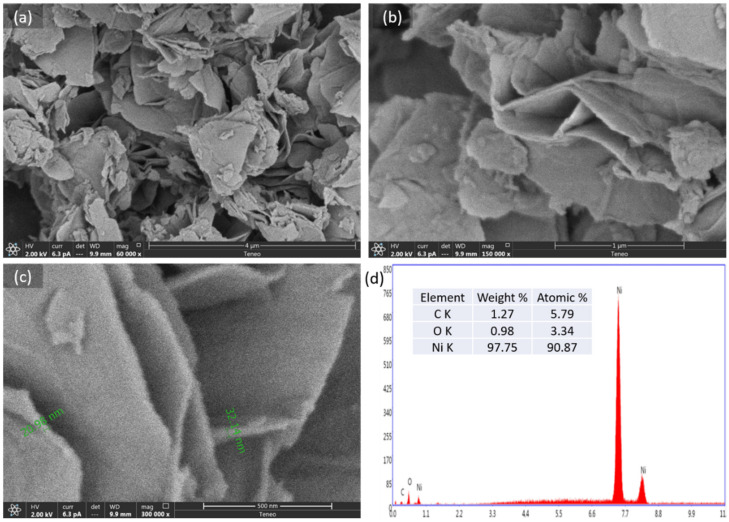
(**a**–**c**) SEM images of N.A.150C.10m at different magnifications and (**d**) the corresponding EDX spectrum with elemental weight and atomic percentages.

Low and high magnified SEM images of the Ni(OH)_2_ powder prepared at 150 °C in 10 min are shown in Figure 2a–c. The Ni(OH)_2_ consists of nanoflake morphology with a flake thickness in tens of nanometers, such as 21 nm and 32 nm. Interestingly, the nanoflake or nanosheet morphology is considered one of the most preferred morphologies for supercapacitor electrodes due to the large possible interface between the electrolyte and the electrode. Furthermore, the array of interconnected Ni(OH)_2_ nanosheets provides abundant cavities and channels for efficient electrons and ions transition, resulting in exceptional electrochemical characteristics. Moreover, the SEM-EDS spectrum detected from the nanoflake in Figure 2d displays the purity of the sample in terms of weight and atomic percentages, confirming the importance of the centrifugation and filtering steps after the synthesis of the samples.

**Figure 3:**
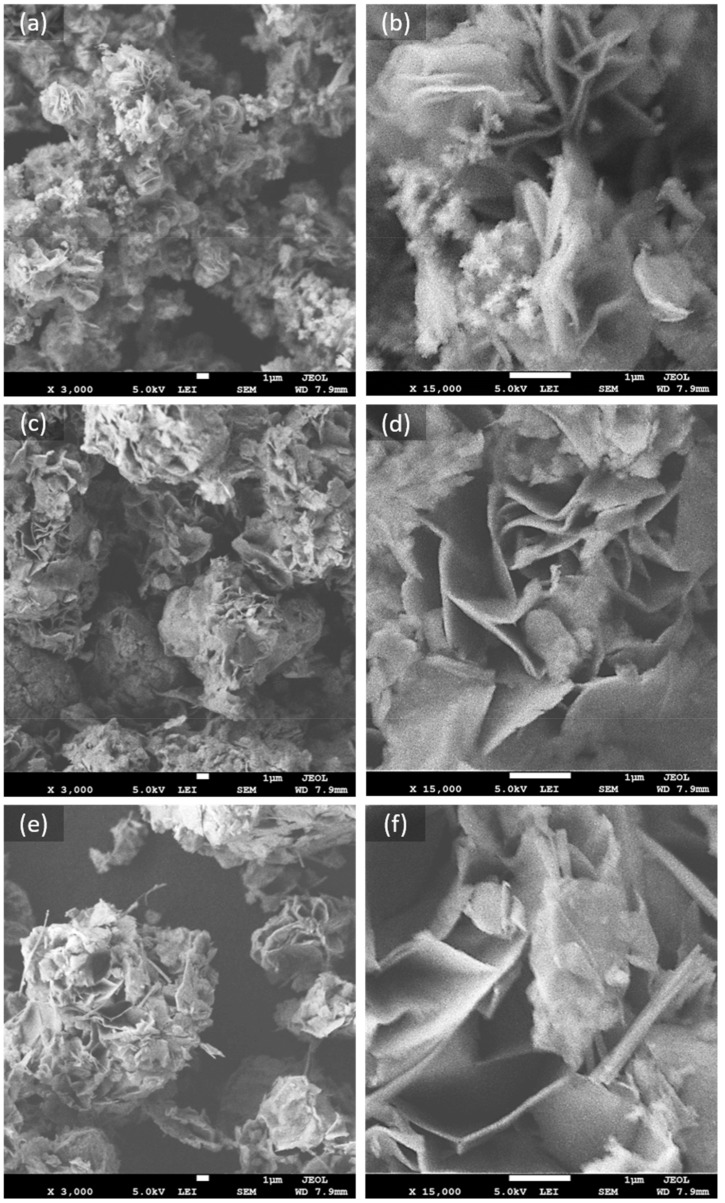
SEM images of (**a**,**b**) N.A.150C.30m, (**c**,**d**) N.A.220C.30m, and (**e**,**f**) N.A.220C.90m, at different magnifications.

Although the nanoflake morphology is successfully obtained at a low temperature of 150 °C in a short time of 10 min, the effects of changing the synthetic conditions on the Ni(OH)_2_ morphology are further examined by SEM images at ×3000 and ×15,000 magnifications. In Figure 3a–c, it can be seen that increasing the temperature and heating time leads to enlarging the diameter of the macro-porous flower-like clusters of interconnected nanoflakes. Nevertheless, the smaller clusters containing the finer nanoflakes in N.A.150C.30m seems to load large material density and hence provide more sites for electrolyte-electrode contact.

### 3.2. Electrochemical Properties

**Figure 4:**
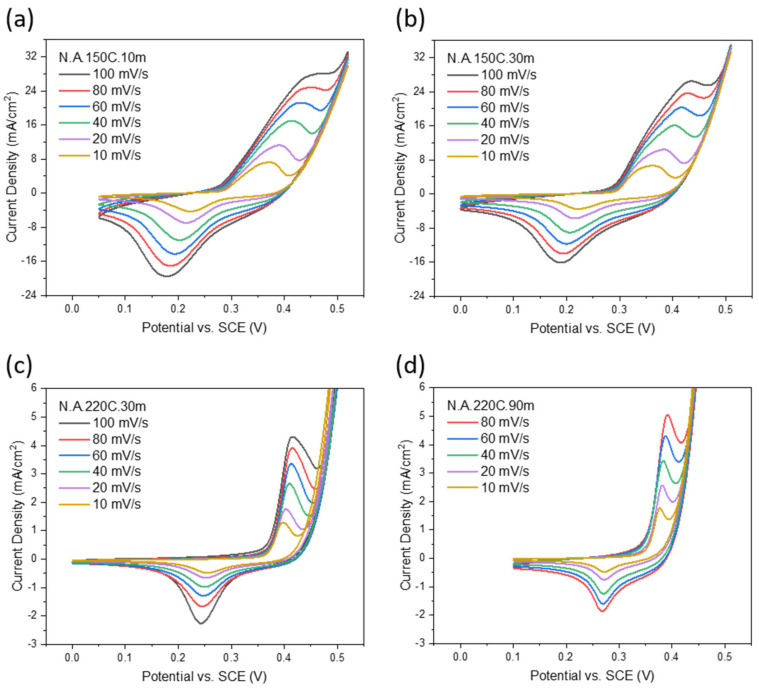
CV curves of (**a**) N.A.150C.10m, (**b**) N.A.150C.30m, (**c**) N.A.220C.30m, and (**d**) N.A.220C.90m electrodes on SS at different scan rates in KOH electrolyte.

For supercapacitor and battery applications, the electrochemical energy storage performance of our Ni(OH)_2_ electrodes on SS substrate is investigated by CV, GCD, and EIS measurements in a 3M KOH electrolyte. Figure 4a demonstrates the effect of applying different voltage scan rates of 10, 20, 40, 60, 80, and 100 mV/s on the CV current response of the N.A.150C.10m electrode. The anodic peaks in the positive current range are caused by the oxidation of Ni(OH)_2_ to NiOOH, whereas the cathodic peaks in the negative current range are caused by the reduction of NiOOH to Ni(OH)_2_. The redox reactions for α-Ni(OH)_2_ electrode are shown in Equation (3) [21,34]:

α-Ni(OH)_2_ + OH^−^ ↔ γ-NiOOH + H_2_O + e^−^ (3)

The redox peaks are still detected up to the high scan of 100 mV/s, implying that the redox reactions of Ni(OH)_2_ can occur even at a fast rate. Additionally, a slight increase in the voltage separation distance between the peaks-pair positions is associated with increasing the scan rate, indicating a quasi-reversible redox reaction.

The electrochemical properties of the N.A.150C.30m, N.A.220C.30m, and N.A.220C.90m electrodes are represented in Figure 4b–d. The current peaks of the N.A.150C.30m electrode are the broadest among the other two samples because this sample contains both α-Ni(OH)_2_ and β-Ni(OH)_2_ phases as resulted from the XRD. Therefore, the N.A.150C.30m electrode is expected to involve double redox reactions in the KOH aqueous electrolyte, as represented in Equations (3) and (4) [21,34]:

β-Ni(OH)_2_ + OH^−^ ↔ β-NiOOH + H_2_O + e^−^ (4)

where the nickel oxidation states difference between +2 in α-Ni(OH)_2_ and +3.6 in γ-NiOOH is larger than the difference between +2 in β-Ni(OH)_2_ and +3 in β-NiOOH [22]. The potential of the oxidation peak at 100 mV/s is 0.435 V vs. SCE, while it is 0.361 V vs. SCE at 10 mV/s. Their potential shift of 0.254 V is more significant than 0.018 V in the N.A.220C.30me electrode and 0.014 V in the N.A.220C.90m electrode. This indicates the electrochemical stability of the mixed-phase N.A.150C.30m electrode is weaker than the pure β-phase electrodes.

**Figure 5:**
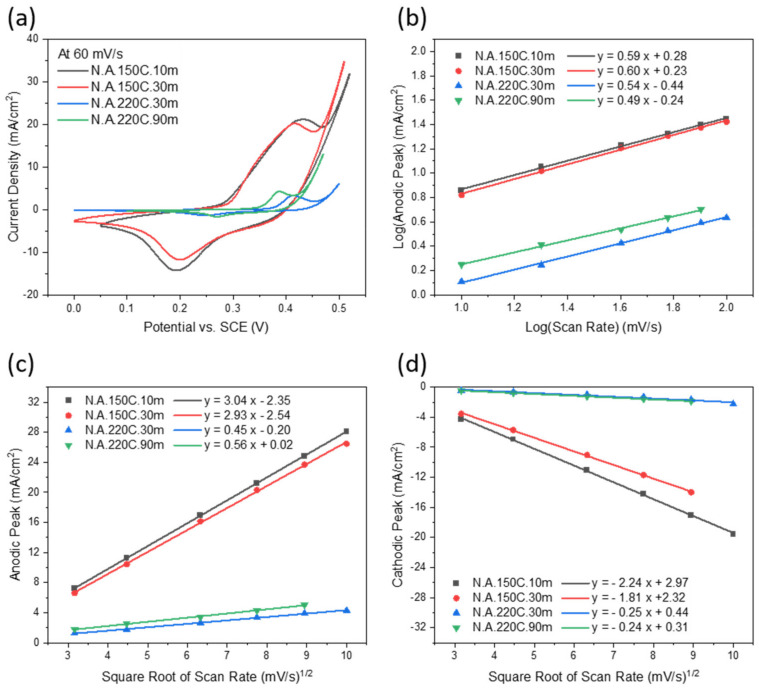
(**a**) CV curves of all electrodes at a scan rate of 60 mV/s; (**b**) the logarithmic anodic peaks vs. the logarithmic scan rates; (**c**,**d**) anodic and cathodic peaks vs. the square root of scan rates, respectively.

Figure 5a compares the previous electrodes’ CV curves at a scan rate of 60 mV/s. It is worth noticing the voltage separation distance between the peak pairs of the N.A.150C.10m and N.A.150C.30m electrodes are broader than that of the N.A.220C.30m and N.A.220C.90m electrodes, representing that the reversibility of the α-based redox reaction is lower than the β-based one. Among all the samples, the N.A.150C.10m electrode exhibits the highest peak current density, similar to the N.A.150C.30m electrode because both electrodes contain α-Ni(OH)_2_. As explained previously, the oxidation state of Ni increased from +2 to +3.6 in the conversion from α-Ni(OH)_2_ to γ-NiOOH, releasing more electrons than the β-phase oxidation reaction [22].

Furthermore, the qualitative determination of the kinetics of the charge-storage mechanism can be distinguished based on the exponent (*b*) of the power-law Equation (5) [42]:

*i*(*v*) = *av^b^* (5)

where (*i*) is the peak current, (*v*) is the scan rate, and (*a*) is a constant. The (*b*) value can be extracted by the slope of the linear relation between *log(i)* and *log(v)*, as represented in Figure 5b. The slop values of ~0.6 for the N.A.150C electrodes and ~0.5 for the N.A.220C electrodes indicate a slow diffusion of electrolyte ions into the electrode’s layers [42,43,44]. In Figure 5c,d, the linear proportion of the anodic and cathodic peaks to the square root of the scan rate (*v*^0.5^) confirms the quasi-reversible diffusion-controlled reactions during the charge and discharge processes [6,43].

**Figure 6:**
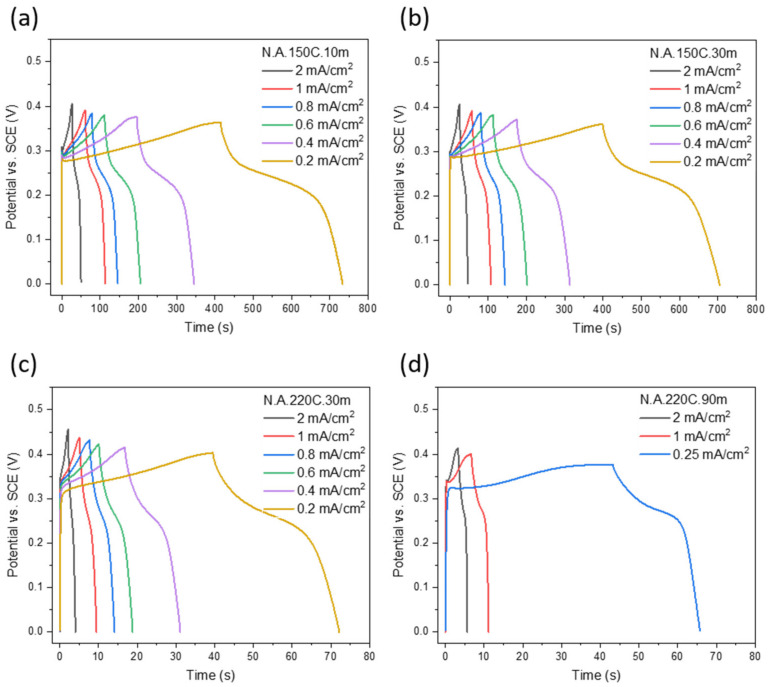
GCD curves of (**a**) N.A.150C.10m, (**b**) N.A.150C.30m, (**c**) N.A.220C.30m, and (**d**) N.A.220C.90m electrodes on SS at different current densities in KOH electrolyte.

The charge–discharge processes are investigated at a wide range of current densities by the GCD curves in Figure 6a–d. The horizontal plateaus in the electrode voltage are produced by redox reactions of Ni(OH)_2_ in alkaline electrolytes, which is a typical characteristic of faradaic capacitors and batteries. By applying small current densities, the charging processes require long times. At high current densities, all electrodes are charged–discharged faster than at low current densities. The longer charge–discharge time of the N.A.150C electrodes than the other electrodes is a consequence of the powerful contribution of the α-phase in the redox reactions.

**Figure 7:**
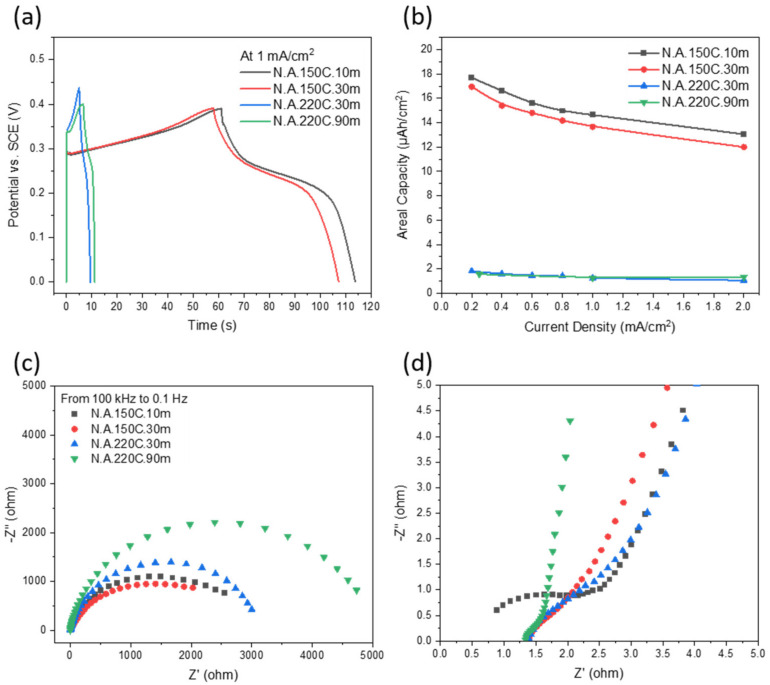
(**a**) GCD curves at a current density of 1 m A/cm^2^; (**b**) areal capacity functions of current densities; (**c**,**d**) Nyquist plots from EIS with enlarged scale.

For the same reason, the GCD curves at 1 mA/cm^2^ in Figure 7a clarify the discharging times of the N.A.150C.10m and N.A.150C.30m electrodes are considerably extended compared to the N.A.220C.30m and N.A.220C.90m electrodes. It is important to remember that the discharge time is directly proportional to the areal capacity. On the one hand, the maximum areal capacities at 0.2 mA/cm^2^ are calculated as 17.7 and 17.0 µAh/cm^2^ for the N.A.150C.10m and N.A.150C.30m electrodes, respectively, significantly exceeded 1.8 and 1.6 µAh/cm^2^ for the N.A.220C.30m and N.A.220C.90m electrodes, respectively. On the other hand, these latter electrodes offer stable areal capacity in the whole range of current density, as shown in Figure 7b. These performance results are expected and supported by the fact that α-Ni(OH)_2_ electrodes have higher theoretical capacities but with lower stabilities than β-Ni(OH)_2_ [39,45]. Experimentally, α-Ni(OH)_2_ flower-like nanostructures prepared by Zhang et al. [38] delivered higher specific capacitance than β-Ni(OH)_2_ nanoparticles reported by Elshahawy et al. [39]. In both cases, the microwave-assisted heating was operating for 30 min, and carbon additives were mixed with nickel hydroxide. The values of current densities, discharge time, areal capacities, and gravimetric capacities for all our additive-free electrodes are listed in Table 3. In comparison, our maximum areal capacity of 17.7 µAh/cm^2^ is higher than 4.5 µAh/cm^2^ at 0.05 mA/cm^2^ for coated Ni–Zn microbattery in 1 M KOH [25], and 4.8 µAh/cm^2^ at 0.04 mA/cm^2^ for NiCoS/Cu@Ni half-cell in 2 M KOH [46]. Table 4 compares the areal capacities of several reported Ni-based electrodes in half- and full-cell confarreations. It can be seen that adding aluminum hydroxide and copper hydroxide to nickel hydroxide improved the electrochemical performance [26]. Moreover, many studies demonstrated the combination of Ni-based composites as battery-type electrodes with capacitor-type electrodes such as reduced graphene oxide [26] or polyaniline [47] for asymmetric hybrid supercapacitors. As shown in Table 4, these asymmetric devices operate in wider voltage windows than the symmetric supercapacitors, utilizing different materials.

Figure 7c,d present the Nyquist plots constructed from the EIS experiments in a frequency range from 100 kHz to 0.1 Hz. In the high-frequency region, the intercept with the *x*-axis provides the value of the resistances of the effective electrode material, the electrolyte, and the current collector, defined as the equivalent series resistance (ESR). Figure 7d clarifies the ESR values of 0.887, 1.400, 1.395, and 1.338 ohms found for the N.A.150C.10m, N.A.150C.30m, N.A.220C.30m, and N.A.220C.90m electrodes, respectively. The smallest value of ESR for the N.A.150C.10m electrode indicates a better conductivity of the α-Ni(OH)_2_. In the low-frequency region, the semi-circles are classified as finite-length Warburg model, caused by short diffusion of the electrolyte’s ions into the electrode [48]. Further developments in the electrode porosity and substrate geometry are recommended to improve the diffusion process.

## 4. Conclusions

In this article, we chose to prepare nickel hydroxide electrodes with the assistance of a microwave oven because it is a simple method that produces high-quality nanomaterials in several minutes and at medium temperatures. Two samples of nickel hydroxide were prepared at heating times of 90 min and 30 min at 220 °C. In addition, we synthesized a third sample of the same substance in 30 min but at a temperature of 150 °C and a fourth sample at the same temperature but in only 10 min. The samples prepared at the highest temperature crystallized in the beta phase of the nickel hydroxide, while the sample synthesized at 150 °C in 30 min contained both beta and alpha phases. Importantly, the pure alpha phase was obtained at 150 °C in only 10 min. Moreover, it was shown that the shape of all the previous four samples is a uniform morphology of interconnected nanoflakes. It can be seen that increasing the temperature and heating time leads to enlarging the diameter of the macro-porous flower-like clusters. Based on that, the temperature of 150 °C and a heating time of 10 min are sufficient parameters for the growth of α-Ni(OH)_2_ nanoflake. Electrochemical measurements in KOH electrolytes demonstrate that electrodes containing the alpha phase have much higher capacities than samples containing only the beta phase, which agrees with published theoretical predictions. The maximum areal capacity of 17.7 µAh/cm^2^ and gravimetric capacity of 35.4 mAh/g were achieved, respectively, at 0.2 mA/cm^2^ and 0.4 A/g for the N.A.150C.10m electrode, with a minimum equivalent series resistance value of 0.887 ohms on flexible stainless-steel mesh as a current collector. These improved results can be ascribed to utilizing the diffusion-controlled redox reactions of α-Ni(OH)_2_ with KOH that are detected from 10 mV/s up to 100 mV/s. Such wide scan rates of charge–discharge processes expand the potential applications. Our nickel hydroxide electrode, with its fast preparation at medium temperature, can be a cost-effective candidate for flexible supercapacitors and batteries.

## Figures and Tables

**Table 1:** Abbreviations of nickel acetate-derived nickel hydroxide samples (N.A.) at different temperatures and times.

Temperature\Time	10 min	30 min	90 min
150 °C	N.A.150C.10m	N.A.150C.30m	---
220 °C	---	N.A.220C.30m	N.A.220C.90m

**Table 2:** The crystallographic structure parameters of Ni(OH)_2_ powder.

Sample	Miller Indices (hkl)	Peak Position (°)	d-Spacing (nm)	Average Crystallite Size (nm)
N.A.220C.90m	(001)	19.1	0.46	4.42
	(100)	33.9	0.26	
	(101)	39.1	0.23	
	(102)	52.7	0.17	
	(110)	59.8	0.15	
	(111)	63.1	0.15	
	(103)	70.5	0.13	
N.A.220C.30m	(001)	19.3	0.46	4.70
	(100)	33.3	0.27	
	(101)	38.7	0.23	
	(102)	52.2	0.18	
	(110)	59.2	0.16	
	(111)	62.7	0.15	
	(103)	70.0	0.13	
	(201)	72.9	0.13	
	(202)	82.7	0.12	
N.A.150C.30m	(001)	11.5	0.77	3.60
	(001)	18.3	0.49	
	(002)	23.4	0.38	
	(110)	33.9	0.26	
	(101)	37.2	0.24	
	(110)	59.8	0.15	
N.A.150C.10m	(001)	11.6	0.76	4.50
	(111)	34.3	0.26	
	(301)	60.2	0.15	

**Table 3:** Electrochemical properties of all electrodes on SS in KOH evaluated from GCD experiments, where the mass loading in each electrode is 0.5 mg/cm^2^.

Electrode	Current Density (mA/cm^2^)	Discharge Time (s)	Areal Capacity (µAh/cm^2^)	Gravimetric Capacity (mAh/g)
N.A.220C.90m	0.25	22.5	1.6	3.1
	1	4.5	1.3	2.5
	2	2.4	1.3	2.7
N.A.220C.30m	0.2	32.6	1.8	3.6
	0.4	14.3	1.6	3.2
	0.6	8.7	1.5	2.9
	0.8	6.4	1.4	2.8
	1	4.4	1.2	2.4
	2	1.9	1.1	2.1
N.A.150C.30m	0.2	305.3	17.0	33.9
	0.4	138.6	15.4	30.8
	0.6	88.9	14.8	29.6
	0.8	63.8	14.2	28.4
	1	49.2	13.7	27.3
	2	21.6	12.0	24.0
N.A.150C.10m	0.2	318.6	17.7	35.4
	0.4	149.6	16.6	33.2
	0.6	93.5	15.6	31.2
	0.8	67.3	15.0	29.9
	1	52.7	14.6	29.3
	2	23.5	13.1	26.1

**Table 4:** Comparative areal capacity and testing conditions of our optimized electrode and some reported Ni-based electrodes.

Electrode/Current Collector//Electrode	Preparation Method (Time)	Electrode Mass/Area	Electrolyte	Voltage (V)	Areal Capacity (μAh/cm^2^)	Current Density (mA/cm^2^)	Ref.
Ni(OH)_2_/SS Half-cell	Microwave-assisted Hydrothermal (10 min)	0.5 mg/cm^2^	3 M KOH	~0.4	17.7 13.1	0.2 2	This Work
NiCoS/Cu@Ni Half-cell	Electrodeposition (30 min) for NiCoS	8 cm^2^	2 M KOH	0.55	4.8 3.8	0.04 5	[46]
NiAl-LDH/Cu Half-cell	Oven-based Co-precipitation (24 h) for NiAl-LDH	~0.61 mg/cm^2^	2 M KOH	0.55	56.5 8.3	5 25	[26]
CH@NiAl-LDH/Cu Half-cell	Solution-immersion (30 min) for CH	~0.85 mg/cm^2^	2 M KOH	0.55	312.4 208.3	5 25	[26]
CH@NiAl-LDH/Cu//rGO/CC Full-cell	Modified Hummer’s Method for rGO	~7.4 mg rGO	2 M KOH	1.55	250	2	[26]
NiCoS/Cu@Ni Symmetric Full-cell	Electrodeposition (30 min) for NiCoS	2 cm^2^	PVA-KOH gel	0.8	1.2 0.4	0.025 0.075	[46]
Ni-Sn/Cu//PANI/Al Full-cell (Nanowire)	Electrodeposition with Pore Template for Ni-Sn (3.5 min)	0.5 cm^2^	1 M LiPF_6_	3.38	3 1	0.03 0.07	[47]
Ni-Sn/Cu//PANI/Al Full-cell (Thin Film)	Electrodeposition for Ni-Sn (5 min)	0.5 cm^2^	1 M LiPF_6_	1.9	~100 ~56	0.2 0.4	[47]
TMV-Ni/Au//Zn Full-cell	Electroless Deposition and MEMS Fabrication	0.64 cm^2^	1 M KOH	0.7	4.5	0.05	[25]

SS: stainless steel, NiAl-LDH: nickel aluminum layered double hydroxide, CH: copper hydroxide, rGO: reduced graphene oxide, CC: carbon cloth, PANI: polyaniline, TMV: tobacco mosaic virus, MEMS: microelectromechanical systems.
